# Whole-Genome Sequences of *Brucella melitensis* Strain QY1, Isolated from Sheep in Gansu, China

**DOI:** 10.1128/genomeA.00896-17

**Published:** 2017-08-31

**Authors:** Xiaoan Cao, Zhaocai Li, Zhongzi Lou, Baoquan Fu, Yongsheng Liu, Youjun Shang, Zhizhong Jing, Jizhang Zhou

**Affiliations:** State Key Laboratory of Veterinary Etiological Biology, Lanzhou Veterinary Research Institute, Chinese Academy of Agricultural Sciences, Lanzhou, Gansu, People’s Republic of China

## Abstract

The facultative intracellular Gram-negative bacterium *Brucella melitensis* causes brucellosis in domestic and wild mammals, and it is a dominant pathogen responsible for human disease. This study reports the whole-genome sequencing of *B. melitensis* strain QY1, isolated from sheep suffering from abortion and arthritis in 2015 in Gansu, China.

## GENOME ANNOUNCEMENT

*Brucella* spp. are Gram-negative and intracellular pathogens that cause animal brucellosis, a disease that leads to abortion and infertility in livestock, resulting in severe economic losses and threats to human health (1, 2). In recent years, brucellosis has reemerged in animals in China. *Brucella melitensis* not only causes the most cases of brucellosis in sheep but is also an important pathogen responsible for human disease (3, 4).

Here, we present the genome sequence of *B. melitensis* QY1, which was isolated in China from the spleen of an aborted sheep fetus. *B. melitensis* QY1 is an epidemic isolate causing typical clinical symptoms (e.g., arthritis and abortion) and histopathologic changes (e.g., significant necrosis in the placenta villi and slight congestion in spleen, with white myeloid lymphocyte density).

Genomic DNA was extracted with the DNeasy blood and tissue kit (Qiagen, Valencia, CA). This genome was sequenced by PacBio technology after PacBio RSII and Illumina HiSeq DNA libraries were constructed to determine the complete genomic sequence of *B. melitensis* QY1. The annotation was performed using GeneMarkS (5), Repeat Masker (6), Tandem Repeats Finder (7), tRNAscan-SE (8), and rRNAmmer (9).

The whole-genome sequence of *B. melitensis* QY1 was found to be 3,311,252 bp in size and composed of two circular chromosomes, with 2,125,648 bp in chromosome I and 1,185,604 bp in chromosome II. The G+C content of this strain is 58.34%. About 86.47% (2,863,398 bp) of the nucleotide sequences are predicted to be coding sequences containing 3,559 genes, and the average gene length is 805 bp. This strain has two small RNAs (sRNAs), 55 tRNAs, and three each of 23S, 16S, and 5S rRNAs.

Functional annotation of *B. melitensis* QY1 genes within the GO database (performed using protein BLAST, E value, ≤1e-5) showed that 2,368 (66.5%) out of 3,559 protein-coding genes had at least some biological function assigned, with some of the genes assigned to more than one category (10).

### Accession number(s).

This whole-genome sequence of *B. melitensis* QY1 has been deposited at DDBJ/EMBL/GenBank under the accession numbers CP022204 and CP022205, and the versions cited in this paper are CP022204.1 and CP022205.1, respectively.
